# Online Depression Communities as a Complementary Approach to Improving the Attitudes of Patients With Depression Toward Medication Adherence: Cross-Sectional Survey Study

**DOI:** 10.2196/56166

**Published:** 2024-11-19

**Authors:** Runnan Chen, Xiaorong Fu, Mochi Liu, Ke Liao, Lifei Bai

**Affiliations:** 1 Department of Marketing School of Business Administration Southwestern University of Finance and Economics Chengdu China; 2 Department of Marketing Business School University of Edinburgh Edinburgh United Kingdom; 3 Education Center for the Master of International Business School of International Business Southwestern University of Finance and Economics Chengdu China; 4 Department of Tourism Management School of Business Administration Southwestern University of Finance and Economics Chengdu China

**Keywords:** online depression communities, attitudes, institution-generated content, user-generated content, perceived social support, antidepressants, hopelessness, cross-sectional study, China, health care system, online health community, depression, medication adherence, social support, health care practitioner, peer support

## Abstract

**Background:**

Lack of adherence to prescribed medication is common among patients with depression in China, posing serious challenges to the health care system. Online health communities have been found to be effective in enhancing patient compliance. However, empirical evidence supporting this effect in the context of depression treatment is absent, and the influence of online health community content on patients’ attitudes toward medication adherence is also underexplored.

**Objective:**

This study aims to explore whether online depression communities (ODCs) can help ameliorate the problem of poor medication taking among patients with depression. Drawing on the stimulus-organism-response and feelings-as-information theories, we established a research model to examine the influence of useful institution-generated content (IGC) and positive user-generated content (UGC) on attitudes toward medication adherence when combined with the mediating role of perceived social support, perceived value of antidepressants, and the moderating role of hopelessness.

**Methods:**

A cross-sectional questionnaire survey method was used in this research. Participants were recruited from various Chinese ODCs, generating data for a main study and 2 robustness checks. Hierarchical multiple regression analyses and bootstrapping analyses were adopted as the primary methods to test the hypotheses.

**Results:**

We received 1515 valid responses in total, contributing to 5 different datasets: model IGC (n=353, 23.3%), model UGC (n=358, 23.63%), model IGC+UGC (n=270, 17.82%), model IGC-B (n=266, 17.56%), and model UGC-B (n=268, 17.69%). Models IGC and UGC were used for the main study. Model IGC+UGC was used for robustness check A. Models IGC-B and UGC-B were used for robustness check B. Useful IGC and positive UGC were proven to have positive impact on the attitudes of patients with depression toward medication adherence through the mediations of perceived social support and perceived value of antidepressants. The findings corroborated the role of hopelessness in weakening or even negating the positive effects of ODC content on the attitudes of patients with depression toward medication adherence.

**Conclusions:**

This study provides the first empirical evidence demonstrating the relationship between ODC content and attitudes toward medication adherence, through which we offer a novel solution to the problem of poor medication adherence among patients with depression in China. Our findings also provide suggestions about how to optimize this new approach—health care practitioners should generate online content that precisely matches the informational needs of patients with depression, and ODC service providers should endeavor to regulate the community atmosphere. Nonetheless, we warn that ODC interventions cannot be used as the only approach to addressing the problem of poor medication taking among patients with severe depressive symptoms.

## Introduction

### Background

It is estimated that there are currently >95 million people with depression in China [1]. Among this population, one-third have never sought professional help, and only 8.99% have taken medicinal treatments [2]. Moreover, among those taking antidepressants, >70% exhibit poor adherence behaviors, meaning that they either arbitrarily alter prescribed dosages or stop taking prescribed medications altogether [3], which will negatively impact the therapeutic outcomes [4]. Such widespread nonadherence poses a serious challenge to the health care system [5]. However, due to the huge population, uneven distribution, and insufficient medical resources in China [6], the amelioration of this problem is extremely tough and tardy.

Fortunately, recent research on online health communities (OHCs) sheds light on a new approach to confronting such dilemmas. OHCs are online platforms that provide patients and health care seekers with access to health care knowledge, experiential information, and social support [7,8]. They can be found in a variety of virtual spaces, such as chat rooms, forums, and bulletin boards on social media or websites [8,9]. Taking advantage of the internet, OHCs can reach a large population of patients at a much lower cost than offline channels [10]. Grounded on this invaluable strength, a great deal of research interest has been paid to investigate OHCs’ potential to address various health care problems. The value of OHCs in enhancing patients’ motivation to adhere to prescribed medications has also been corroborated. Yin et al [11] determined that OHC member reciprocity positively affected patients’ completion of a 5-year regimen. Wu et al [12] found that informational support from OHCs strengthens patients’ trust in health care services and boosts their adherence intentionality. Lu and Zhang [13] and Lu [6] revealed that the physician-patient relationship, along with patients’ health information–seeking behaviors, becomes better in OHCs, resulting in patients’ increasing willingness to comply with physicians’ instructions. These findings ascertain that OHCs can be an effective tool to remediate patients’ attitudes toward medication adherence, thereby refining the nonadherence problem.

However, depression has some distinctions compared to general diseases. Patients with depression experience extensive impairments in cognition, well-being, and social life, putting them at high risk of self-harming and even suicide owing to overwhelming agony and desperation [14]. Statistical results have also revealed that a poor medication adherence problem is much more prominent and prevalent among patients with depression [2,3,5]. In line with such uniqueness, online depression communities (ODCs), which are communities exclusive for stakeholders in depression treatment (eg, patients, physicians, and psychological counselors) to discuss depression-related topics, have prosperously developed in cyberspaces and are usually separated from OHCs in academia [15-18]. Therefore, we are concerned that the conclusions of previous OHC research might not necessarily hold true in the depression treatment and ODC context. Additional empirical studies to verify ODCs’ influence on the attitudes of patients with depression toward medication adherence are warranted.

Furthermore, previous research on the relationship between OHCs and medication adherence has primarily treated users’ participation behaviors or perceptions of the overall OHC environment as determinants of their received benefits, with little emphasis placed on the role of community content. Serving as the touch point between an online community and its members, community content is closely related to how users will be influenced by the community [19]. In particular, Nimrod [20] verified that the influence of ODC content is not homogeneous—content with different features will affect recipients in different ways. This finding suggests the importance of taking the characteristics of the content into consideration when examining ODCs’ impact. Therefore, this study intended to explore the value of ODCs in improving the medication adherence attitudes of patients with depression from a community content perspective.

### Theoretical Foundation

#### Stimulus-Organism-Response Theory

ODC content can be viewed as a series of external stimuli to community users, and variation in attitudes toward medication adherence is a potential user response. Therefore, we introduce the stimulus-organism-response (SOR) theory [21] to ascertain the relationship between ODC content and attitude toward medication adherence. Following the environmental psychology perspective, SOR theory states that external stimuli affect human behavior by influencing an individual’s internal experience (organism), thereby eliciting a response [21]. Its validity in theorizing the influence of online communications has been corroborated by previous literature. In particular, several studies have used this theory to evaluate the relationship between OHC characteristics and the responses of community members. Their results indicate that users’ positive responses are attributable to positive information dissemination within the community, such as emotional support, high-quality content, and an enjoyable atmosphere [7,22,23]. In contrast, negative informational factors, such as information overload and system functionality overload, lead to resistant behavior [24]. On the basis of these findings, we postulate that favorable stimuli evoke affirmative responses, whereas unfavorable stimuli prompt adverse responses. The improvement in medication adherence attitudes is conceivably the result of stimulation of ODC content carrying positive features.

Moreover, SOR theory offers a well-established paradigm to explore the inner mechanism through which patients with depression are influenced by ODC content using a series of psychological activities [25]. On the basis of this theoretical framework, we assume that patients’ attitudes toward medication adherence might be improved concurrently with alterations in some subjective factors that closely relate to the adherence to antidepressants, initially through the stimulation of specific ODC content. Previous studies have identified that psychological activities that directly affect patients’ willingness to adhere to antidepressant treatment primarily include their perceived support from their social network and their subjective evaluations of the pros and cons of taking antidepressants [26-29]. These mental processes can be boiled down to the concept of perceived social support and perceived value of antidepressants. Both are perception-level factors that can be influenced by information disseminated through online communities [6,30]. Therefore, we predict that they may serve as mediators between the extrinsic stimuli that patients receive from ODCs and their responses.

#### Feelings-as-Information Theory

Nevertheless, as this study strives to understand how patients with depression, which is a type of mood disorder [14], perceive and respond to external stimuli, it is necessary to take their emotional states into consideration. The SOR theory is valuable for postulating connections between ODC content stimuli and responses in patients with depression. However, it does not explain how mental state as a kind of internal information filter influences individuals’ decision patterns. Hence, we incorporate the feelings-as-information theory [31] to cover this deficiency and aid in enhancing the overall integrity of our theoretical framework. The feelings-as-information theory is a critical theory in emotion research. It delves into how subjective feelings, such as emotions, moods, and metacognitive experiences, impact human judgments [31-33], offering a comprehensive model to describe the interplay between emotion and thought. According to this theory, an individual’s state of mind affects their processing style when dealing with external information and, thus, impacts their judgments [31,32]. Its effectiveness in interpreting how feelings moderate people’s responses to information on social media has also been confirmed by previous literature [34-36]. As the motivation to adhere to antidepressant treatment is a symbol of trying to save oneself from depression, we surmise that hopelessness—a state of mind in which people lose heart and cease to care about life or the future [14]—will play a key role in moderating the positive effect of ODC content on patients’ attitudes toward medication adherence.

### Research Hypotheses

#### The Content in ODCs

In essence, all informational materials within an online community are generated by its participants, whereas the information from different content providers may be heterogeneous [18,19]. Typically, the discrepancy in specialized knowledge and expertise can differentiate participants’ roles in an online community [19]. Health care providers in particular can educate other users (eg, patients) by sharing professional health information [37,38]. ODCs are technically communities that address health-related issues. Thus, when studying the influence of ODC content, distinguishing between institution-generated content (IGC) and user-generated content (UGC) is essential.

IGC refers to content posted on online communities by specialized agencies, such as governmental agencies, hospitals, or psychology institutes, and personnel associated with those agencies [39]. Researchers have learned that it is common for OHC users to create or disseminate inaccurate or even false information in the communities, which may mislead other platform users [39,40]. IGC is generally considered more credible, authoritative, and trustworthy than content created by lay users [41,42]. When seeking specific information about a condition or disease, people are inclined to go to a reputable source [39]. Nevertheless, studies have shown that, despite the voluminous content in ODCs, individuals with depression still have questions and unmet informational needs [15]. Therefore, we hypothesize that the perceived usefulness of IGC may be a significant factor affecting IGC’s influence on content recipients.

UGC is content created by users in the community [18]. Due to the anonymity within ODCs and the fact that all participants experience depression, community members come together in a safe space to communicate their experiences of living with depression and exchange sympathy or encouragement with others [8,40]. While this content is generally not as accurate and reliable as IGC [39,41,42], it provides ODC users with experiential knowledge about depression treatment [16] and emotional support [18], which cannot be accessed through IGC. However, the tone of UGC in ODCs is usually mixed because users come to the sites in a depressed state [16]. In addition to content that offers positive support, ODCs are rife with negative UGC that conveys anguish and despair [16]. Research has shown that individuals who experience depression may be less likely to seek assistance if they perceive the received messages related to depression as negative [17,43]. Therefore, we firmly believe that perceived positivity is a decisive factor in influencing UGC’s ability to help patients with depression improve their attitudes toward taking medications.

Overall, we extracted 2 types of content carrying positive features in the ODC setting: useful IGC and positive UGC. In our notion, they can serve as positive extrinsic stimuli for patients with depression, eliciting improved attitudes toward taking medications. Useful IGC provides needed and reliable health information to patients [37,38]. This can enrich their knowledge about depression and antidepressants, through which irrational concerns about the side effects of medications can be alleviated and facts about the purpose and value of medications can be clarified, making patients more inclined to adhere to prescribed medicinal protocols [28,29]. Positive UGC, on the other hand, provides opportunities for community members to communicate with other patients with depression about their experiences of coping with depression [16,40]. Such positive content can alleviate their loneliness during treatment and empowers them to fight depression more actively [9]. Moreover, it helps patients understand their disease and evaluate medical treatments based on the experience of others with the same condition [8,44]. Gradually, as they develop a more comprehensive view of antidepressant treatment, they may have a more positive attitude toward medicinal treatment and become more willing to take antidepressants [27,45,46]. Consequently, we propose the following:

- The usefulness of IGC has a positive effect on the attitudes of patients with depression toward medication adherence (hypothesis 1a).
- The positivity of UGC has a positive effect on the attitudes of patients with depression toward medication adherence (hypothesis 1b).

#### The Mediating Role of Perceived Social Support and Perceived Value of Antidepressants

##### Perceived Social Support

Social support is defined as the exchange of resources between individuals who play the roles of support provider and support recipient with the function of improving the recipient’s well-being [47]. Feldhege et al [18] determined that the communications within ODCs can primarily provide 2 types of support to users: informational support and emotional support. In line with this finding, both IGC and UGC can be potential social support resources for ODC members. On the one hand, IGC is a source of authoritative health care information [39], consisting of reliable messages that cover topics such as the disease, treatments, health care services, and coping strategies for depression. They can offer informational support to individuals with depression, helping them gain a better understanding of depression and how to deal with it [15,18]. In contrast, UGC refers to the communications among community users. Social discrimination and lack of public understanding of depression are significant issues in China, causing individuals with depression to be marginalized and ostracized [17]. Through ODCs, patients with depression connect and relate to others in similar situations [40], providing mutual help and mitigating individual loneliness [9]. In this way, UGC generates potential emotional support for ODC participants [40].

Nevertheless, perceived social support is to some extent different from received social support [48]. In addition to availability, perceived social support is also significantly influenced by recipients’ satisfaction with the support provided [49]. To positively influence perceived social support, the support provided needs to match people’s needs as it is only when individuals feel that their issues are successfully addressed or mitigated that they perceive the benefit of social support [50,51]. Grounded on this rationale, not all ODC content can uplift users’ perceived social support. For instance, IGC that is unhelpful to users and UGC that discusses negative topics (eg, self-harm, suicide, or despair) cannot make the recipients feel well supported. Therefore, we argue that the usefulness of IGC and positivity of UGC are important driving factors for perceived social support. Useful IGC fulfills users’ specific demands for knowledge about depression and coping strategies, whereas positive UGC soothes users’ loneliness and agony, thereby enhancing their perceived social support. Accordingly, we propose the following:

- The usefulness of IGC has a positive effect on the perceived social support of patients with depression (hypothesis 2a).
- The positivity of UGC has a positive effect on the perceived social support of patients with depression (hypothesis 2b).

Patients with depression are frequently pessimistic and unmotivated [14], making them likely to avoid challenges and stop taking prescribed medications [5]. In this case, they might need some additional support to encourage them not to lose hope of recovering. If they perceive strong social support when they are tempted to stop taking their medications, they are reminded that they are not alone and there are others who understand their misery. Thereby, they can be empowered to keep fighting depression [9]. Consistent with this rationale, clinical studies have concluded that patients with depression with strong social support systems generally have better attitudes toward medicinal interventions and are less likely to abandon treatment ahead of schedule [4,26]. Therefore, we propose the following:

- Perceived social support has a positive effect on the attitudes of patients with depression toward medication adherence (hypothesis 3).

##### Perceived Value of Antidepressants

Perceived value is generally determined by consumers’ subjective evaluations of a product’s potential benefits and costs [52,53]. As the evaluation process is not objective, perceived value may significantly differ from a product’s actual value due to consumers’ lack of understanding of the product or excessive concerns about its potential risks [52,54]. Such a phenomenon is typically salient in the setting of antidepressant treatment. Although clinical research has verified that all first-line antidepressants have substantial efficacy in improving depressive symptoms [55] and most side effects recede spontaneously after an initial phase of use [56], a misconception that taking antidepressants is painful and ineffective still widely exists, negatively impacting patients’ perceived efficacy and willingness to adhere to antidepressant treatment [27-29,57].

Fortunately, perceived value is a dynamic trait [58]. As consumers gain more exposure to a product, their evaluation of its value tends to become more comprehensive [59]. Irrational concerns about a product’s potential risks can also lessen as consumers learn more about it [60]. In line with these findings, clinical research has confirmed that objective knowledge about antidepressants is positively associated with patients’ beliefs about their value and willingness to use them [45,46]. Through the IGC channel, patients with depression can conveniently access information about antidepressants and obtain specific details about their features and functions curated by health experts [37,38]. When users perceive that the information presented is believable and useful, the information is stored in their memory and serves to enhance their objective knowledge of the medicines [61]. With more objective knowledge, they better understand the benefits, and their concerns about potential adverse reactions to medications are allayed, both significant factors influencing perceived value. Therefore, we hypothesize the following:

- The usefulness of IGC has a positive effect on patients with depression’s perceived value of antidepressants (hypothesis 4a).

UGC has also been found to affect consumer attitudes and value perceptions about products discussed in online communities through peer influence [62]. Peer influence occurs among a group of people who share common characteristics or interests and is actually a mechanism of learning by imitation [63]. Individuals may absorb and internalize information generated by their peers in a conscious or unconscious way, leading to a convergence of group members’ opinions and beliefs [63,64]. Relevant studies have also revealed that peers’ experiences of a medical treatment will influence patients’ judgments on its benefits [44,46,64]. Therefore, we postulate that, when patients are exposed to UGC that relates positive personal experiences of antidepressant treatment, they may internalize the information and develop a more positive perception of antidepressants. Accordingly, we propose the following:

- The positivity of UGC has a positive effect on patients with depression’s perceived value of antidepressants (hypothesis 4b).

The relationship between the perceived value of antidepressants and attitudes toward medication adherence is fairly obvious. A high perceived value indicates that consumers believe that using antidepressants can bring more benefits than costs [52], and therefore, they are willing to purchase or use these medications [53]. These patients understand that taking antidepressants can help ease or even alleviate their depression, and this understanding is significantly related to their attitudes toward medication adherence [65]. Hamrin et al [5] also noted that, when patients with depression believe that antidepressants are helpful to them, their intrinsic motivation to comply with treatment is substantially enhanced. Therefore, we suggest the following:

- Perceived value of antidepressants has a positive effect on the attitudes of patients with depression toward medication adherence (hypothesis 5).

##### Mediation Effects

In line with the SOR theory, an individual’s organism, which may be any internal assessment correlated with or aroused by a stimulus, is a key mediator between the external stimuli and the response [25]. The feasibility of applying this theory in the depression treatment context is reinforced by clinical research outcomes. As a pharmacological treatment for a mental disease, the effectiveness of antidepressants is revealed more by patients’ self-reporting than by physiological indicators [57]. Whether medical treatments are effective and whether they merit continuation depends largely on patients’ subjective evaluations. Consequently, the willingness to accept medicinal interventions has been confirmed to be closely associated with a variety of cognitive factors [3,27-29,65]. Therefore, it is reasonable to theorize that patients’ internal experiences play a significant mediation role in our research setting.

Perceived social support is an individual’s feeling about whether there is someone able and ready to provide help when they are in need [48]. Perceived value is a subjective evaluation of a product’s benefits and costs [52]. They are both subjective perceptions and, thus, meet the criteria to be regarded as organisms [25]. Therefore, we propose that perceived social support and perceived value of antidepressants play mediating roles between ODC content stimuli and the attitudes of patients with depression toward medication adherence. As patients are exposed to useful IGC that enables them to better cope with the challenges of depression and the medication process or positive UGC about others’ experiences of fighting depression, perceived social support and perceived value of antidepressants increases, and this in turn boosts their attitudes toward adherence to medicinal treatment. Accordingly, we propose the following hypotheses:

- Perceived social support mediates the effect of the usefulness of IGC on the attitudes of patients with depression toward medication adherence (hypothesis 6a).
- Perceived social support mediates the effect of the positivity of UGC on the attitudes of patients with depression toward medication adherence (hypothesis 6b).
- Perceived value of antidepressants mediates the effect of the usefulness of IGC on the attitudes of patients with depression toward medication adherence (hypothesis 7a).
- Perceived value of antidepressants mediates the effect of the positivity of UGC on the attitudes of patients with depression toward medication adherence (hypothesis 7b).

#### The Moderating Role of Hopelessness

In line with the feelings-as-information theory, individuals’ moods have substantial influence on their processing styles when dealing with external information [31]. In particular, Schwarz et al [66] ascertained that a negative affect can act as an alarm bell, alerting people that they are in a problematic situation. Such information provides cognitive tuning to adapt a person’s processing strategies to situational requirements [66]. As having bad moods is an obvious symptom of depression [14], practically all patients with depression satisfy the condition for this effect. However, the negative emotions that patients with depression bear may continue to escalate and become extremely serious. Marchetti [67] noted that people trapped in depression and misery for a long time will develop a sense of hopelessness. With such an emotional state, even when offered the possibility of escape, they choose to remain in the status quo rather than try to alleviate their symptoms [68]. Consistent with this rationale, clinical studies show that poor medication adherence is frequently exhibited by patients with intense negative moods in the treatment of chronic diseases such as HIV or diabetes [69-71]. Hosek et al [71] and DiMatteo et al [72] have suggested that the underlying cause is a feeling of hopelessness that often accompanies severe depressive symptoms, which leads patients to believe that their actions will not bring desired outcomes [67] and extinguishes their motivation to adhere to treatments [72].

Collectively, these findings suggest that a negative emotional state, which can range from mild sadness to intense hopelessness, is closely related to patients’ decision patterns in the medication treatment process. Nevertheless, it should be noted that, the current rate of uptake and general adherence to antidepressants in extremely low around the globe [2-5,29]. Skepticism about the medication’s purpose can arise due to lack of understanding [27,29]. It is common for those with depression to struggle with sticking to their medication routine, often due to lack of motivation and positive reinforcement [5]. Even emotional state notwithstanding, they may still have trouble trusting that the medication will work for them. Therefore, we propose that hopelessness, rather than directly determining motivation to adhere to antidepressant treatment, may serve as a significant factor regulating the influence of positive intervention on the medication adherence attitudes of patients with depression. Our hypothesis suggests that the use of positive ODC stimuli can play a crucial role in improving the willingness of patients with depression to adhere to their medication routine. We theorize that hopelessness serves as a key moderator of the relationship between a patient’s attitude toward medication adherence and the positive stimuli from ODCs.

More specifically, patients who are hopeful about their lives and futures will be driven to deal with their depression by actively exploring coping strategies [66]. With useful IGC that offers authoritative knowledge about antidepressants and positive UGC that depicts successful recovery experiences, patients with depression may come to understand that antidepressants can help them and be inspired to adhere to treatment. However, when patients reach a certain level of hopelessness, they become overwhelmed by the negative belief that any recovery attempts are useless [67]. With such biased cognition, they remain mired in the status quo and cannot be persuaded to comply with the medicinal treatments even when provided with useful IGC and positive UGC as supports [68,72]. Consequently, we propose the following hypothesis:

- Hopelessness negatively moderates the effects of the usefulness of IGC and the positivity of UGC on the attitudes of patients with depression toward medication adherence such that the effects are stronger when a patient has a low level of hopelessness but weaker or even negated when a patient has a high level of hopelessness (hypothesis 8).

### Research Model

**Figure 1:**
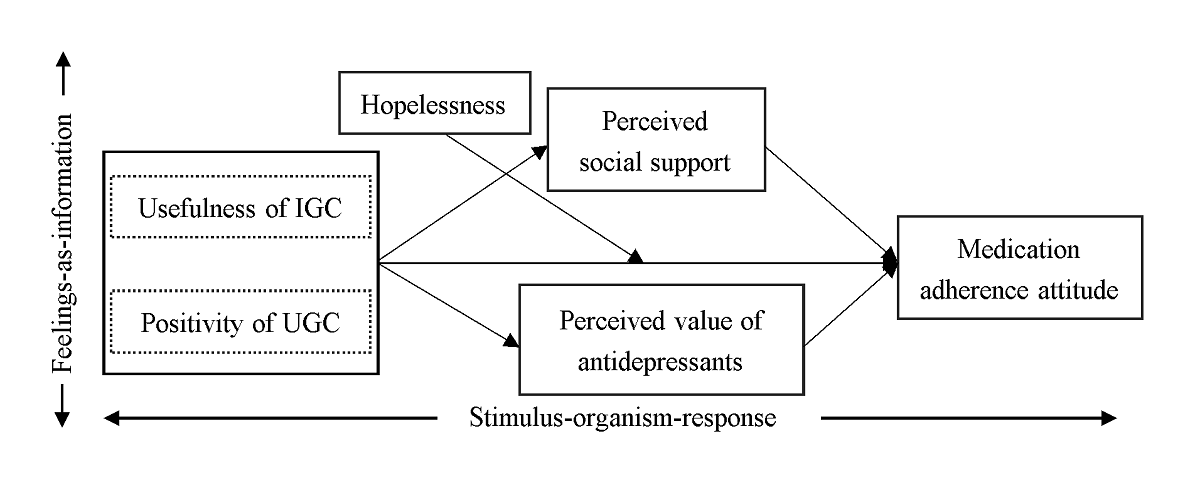
Research model. IGC: institution-generated content; UGC: user-generated content.

Overall, under the guidance of the SOR and feelings-as-information theories, this study established a research model (Figure 1) theorizing the influence of useful IGC and positive UGC on the attitudes of patients with depression toward medication adherence considering the mediating effects of perceived social support and perceived value of antidepressants and the moderating role of hopelessness. This study will provide the first empirical evidence demonstrating whether ODCs can be used as an effective complementary approach to addressing the nonadherence problems among patients with depression, and it will contribute to the preliminary knowledge on its inner mechanism and boundary conditions.

## Methods

### Study Design

This research aimed to explore the influence of ODC content on the willingness of people with depression to adhere to medicinal treatments. A main study and 2 robustness checks, all of which used a cross-sectional survey method, were designed to test our hypothetical model.

The main study was conducted to examine our research model using data collected from patients with depression who had only read one type of ODC content (ie, IGC [content provided by professional institutions] or UGC [content provided by other patients with depression]). The previous work by Lu [6] revealed that, as the specialization of OHCs develops, some OHCs exclusive for patient-to-physician communications or patient-to-patient communications are being established in China. Under such circumstances, some ODC users have only been exposed to either IGC or UGC. Volunteers who had only read one type of ODC content were less likely to experience the distraction of recalling another type of content, generating more plausible data. Thus, we used the data collected from them (named model IGC and model UGC) for the main study to verify our hypotheses.

Robustness check A, whose data (named model IGC+UGC) were collected from volunteers who had been exposed to both types of content, was implemented to optimize the generalizability of our research findings. This substudy validated whether the effects of useful IGC and positive UGC could function simultaneously and examined whether they would impact patients with depression differently.

Robustness check B, whose data (named model IGC-B and model UGC-B) were also collected from volunteers who had only read one type of ODC content, was conducted to further corroborate our core hypotheses—the effects of ODC content on the attitudes of patients with depression toward medication adherence. In this substudy, participants’ ODC use duration and frequency were additionally measured to evaluate how the influence of IGC usefulness and UGC positivity on participants’ medication adherence attitudes varied along with their ODC use situations. This study was designed to reduce the likelihood that the identified effects of ODC content on medication adherence attitudes were false-positive results.

### Data Collection and Respondent Profile

We used convenience sampling and snowball sampling in our data collection process. Given that users from the same ODC likely have similar perceptions about their community as they are exposed to the same information, we did not rely on a single ODC to obtain data for our research. Instead, we posted research advertisements on several health care websites (eg, “SunForum”), online forums (eg, “Baidutieba”), online depression group chats (eg, “Depression Tree Hole”), and social media platforms (eg, “Xiaohongshu”) to recruit participants from various ODCs. Meanwhile, we encouraged participants to share our questionnaire with their peers with depression who were also using ODCs. The sample recruitment advertisement is provided in Multimedia Appendix 1.

Because previous research has found that approximately one-third of individuals with depression in China have never sought professional help and, therefore, have not been officially diagnosed with depression [2], we sought to include this significant cohort in our study population [9]. Both patients with diagnosed depression and self-reported depression were targeted so that our sample reflected the population of individuals with depression in China as a whole, ensuring that our data were not biased and the validity of the study was not impugned.

The general inclusion criteria for this study were that the participants must currently have depression and be users of ODCs. Participants were regarded as experiencing depression if they met one of the following conditions: (1) they had been officially diagnosed with depression by a physician and had not yet recovered (diagnosed depression), (2) they had been suggested to have a high likelihood of being depressed by a self-rating depression scale and believed that they were still in a depressive status (self-reported depression), or (3) they had experienced depression-related symptoms for a rather long time and strongly believed that they might have depression (self-reported depression). The scope of ODCs was defined as “digital communities exclusive for stakeholders in depression treatment (eg, patients, physicians, and psychological counselors) to discuss depression-related topics.” The additional criterion for each substudy, which related to the type of ODC content that the participants had read, is provided in the Study Design subsection. Participants were excluded if they reported no depression experience, had already recovered from depression, or were not using ODCs.

To mitigate the limitations of self-reported data, the following steps were taken. First, the survey was anonymous, and we emphasized to the participants that all information they provided to us would be kept strictly confidential and only used for research purposes. This helped reduce the risk of social desirability bias by mitigating participants’ privacy concerns and fear of judgment [73]. Second, we repeatedly reminded the participants throughout the questionnaire that the accuracy of their responses was vital to the success of this study and the common well-being of the population of patients with depression. In this way, we sought to encourage participants to allocate more cognitive resources to our study and answer the questions more carefully, honestly, and precisely [74]. Third, we manually scrutinized every response received. Participants whose responses displayed obvious signals of carelessness (eg, answering all questions with the same choice, random answers, and very short response time) were excluded from our samples [75].

The whole data collection fieldwork was conducted in 3 periods. The first period was from June 2022 to September 2022, which collected 469 effective responses for our main study. The second period was from July 2023 to August 2023, generating 173 effective responses for robustness check A. The third period was from April 2024 to June 2024, providing 873 effective responses for our main study, robustness check A, and robustness check B. Overall, 711 effective responses for the main study (n=353, 49.6% for model IGC and n=358, 50.4% for model UGC), 270 effective responses for robustness check A (all for model IGC+UGC), and 534 effective responses for robustness check B (n=266, 49.8% for model IGC-B and n=268, 50.2% for model UGC-B) were collected. Of the overall respondents, 67.1% (1017/1515) were patients with diagnosed depression, and 32.9% (498/1515) were patients with self-reported depression, closely mirroring the statistics provided by Yu et al [2]. Multimedia Appendix 2 provides more detailed sample characteristics.

### Ethical Considerations

This study received ethics approval from the School of Business Administration of Southwestern University of Finance and Economics. Informed consent was obtained from all participants and was necessary for submitting the digital questionnaire. The questionnaire was anonymous and did not collect any identifying information, such as name or address. Eligible participants were given CN ¥10 (US $1.40) in cash as compensation.

### Measurement Instruments

#### Overview

The measurements for each construct were adapted from existing research and modified based on the context and the target audience. Specifically, in the preliminary stage, we reviewed related literature to obtain the seminal scales of reference variables. All items that were originally in English were translated into Chinese and cross-culturally adapted. In addition, we interviewed 5 patients with depression to contextualize the items. Next, 2 psychology experts, 2 marketing experts, and 2 medical experts conducted reviews to refine the wording of the instrument items. Their feedback provided the basis for revising the construct measures and modifying wording and item sequence. Finally, we back translated the Chinese questionnaire into English to ascertain its accuracy, completeness, and consistency compared to the original text. The full measure items are listed in Multimedia Appendix 3. All theoretical variables were measured on 7-point Likert-type scales ranging from 1 (*strongly disagree*) to 7 (*strongly agree*). The descriptive statistics and reliability of each variable are shown in Multimedia Appendix 4.

#### Usefulness

The perception of IGC usefulness was measured using a 3-item scale developed by Lee and Hong [76].

#### Positivity

We used a 4-item scale to measure the perception of UGC positivity. The scale was adapted from the friendliness scale by Price and Arnould [77] in accordance with the definition of positive feedback by Fong et al [78].

#### Perceived Social Support

We measured perceived social support using an 11-item scale adapted from the Multidimensional Scale of Perceived Social Support by Zimet et al [79]. The scale by Zimet et al [79] originally measured 3 dimensions: perceived support from significant other, perceived support from family, and perceived support from friends. This multidimensional measurement style is inapplicable in the ODC context. Thus, we adapted this scale, making it a unidimensional scale measuring the comprehensive perception of support from the overall society.

#### Perceived Value of Antidepressants

The perceived value of antidepressants was measured using a 5-item scale developed by Sweeney and Soutar [80].

#### Medication Adherence Attitude

Medication adherence attitude was measured using a 3-item scale adapted from the Adherence Attitude Inventory developed by Lewis and Abell [81].

#### Hopelessness

We used a 17-item scale adapted from the Hopelessness Scale by Beck et al [82] to measure the current hopelessness intensity of patients with depression.

#### ODC Use Duration and Frequency

In robustness check B, participants’ ODC use duration and frequency were measured. Participants’ ODC use duration was measured by calculating the interval between the reported date of their first visit to the ODC and the date when they responded to our survey. Participants’ average ODC use frequency was measured using 10 options ranging from “once a month” to “at least once a day.” Each option was converted into a visit per day form for statistical analyses; for example, “once a month” was counted as 0.0333 visits per day (1 visit per 30 days), “once a week” was counted as 0.1428 visits per day (1 visit per 7 days), and “at least once a day” was counted as 1 visit per day.

#### Control Variables

Following previous research [9], we chose participants’ mental health condition, gender, age, educational level, and marital status as the basic control variables in our study. Given that collectivism is strongly emphasized in China, especially within the family of origin [17], and that the medication adherence situations of patients with depression are closely associated with their families of origin [3], we decided to include family of origin as a control variable. Furthermore, relevant research suggests that recipients’ perception of the credibility of online health information influences its communication effect [39]. To eliminate the interference of this factor, we additionally included the perceived credibility of IGC and UGC as a control variable using a 4-item scale developed by Rimmer and Weaver [83] to measure it.

### Assessment of Measurement Validity

#### Common Method Bias Test

Given that all variables in our study were self-reported by participants, our research might be affected by common method bias [84]. We conducted the single-factor test by Harman [85] to examine the underlying seriousness of common method bias. In the main study, for items in model IGC, 7 factors with an eigenvalue of >1 were extracted, with the first factor accounting for 22.67% of the variance; for the items in model UGC, 6 factors with an eigenvalue of >1 were extracted, with the first factor accounting for 23.56% of the variance. In robustness check A, 9 factors with eigenvalues of >1 were identified, with the first factor accounting for 18.45% of the variance. In robustness check B, 3 factors with an eigenvalue of >1 emerged from model IGC-B, with the first factor explaining 33.01% of the variance; 4 factors with an eigenvalue of >1 emerged from model UGC-B, with the first factor explaining 24% of the variance. As the first factor in each model only accounted for a small proportion of the total variance and several factors emerged, common method bias did not appear to be a serious problem for our research [84].

#### Convergent Validity and Discriminant Validity

Convergent validity is considered acceptable if the value of the standardized factor loading of each item is >0.5 and the composite reliability (CR) and average variance extracted (AVE) of each variable are >0.7 and >0.5, respectively [86]. Confirmatory factor analysis was conducted, and the results showed that the standardized factor loadings of all variables were >0.5. Corresponding CRs and AVEs computed based on the standardized factor loadings are shown in Multimedia Appendix 5. All CR and AVE values satisfied the required standards, ensuring convergent validity. Discriminant validity differentiates a construct from other constructs in the same model. A classic method to assess discriminant validity is comparing the square root of the AVE and the correlation between 2 constructs [87]. As shown in Multimedia Appendix 5, the square root of the AVE for each variable in our models was greater than its correlations with other variables. Therefore, discriminant validity was confirmed.

## Results

### Results of the Main Study

#### Assessment of Model Fit

Before verifying the proposed hypotheses, we needed to confirm whether the hypothesized model fit the data well. While 2 of the latent variables in our models, perceived social support (11 items) and hopelessness (17 items), were measured using a great number of items, a large parameter estimation bias might be incurred if we directly used the original items to construct the models. To avoid this interference, previous research has suggested parceling the scales if they are unidimensional [88]. Both the scales of perceived social support and hopelessness used in this study fulfilled this requirement. Therefore, we parceled them into 3 items each following the factorial algorithm proposed by Rogers and Schmitt [89]. The modified models’ fit analyses yielded the following results for model IGC—χ^2^/df=2.0, *P*<.001, comparative fit index (CFI)=0.977, Tucker-Lewis index (TLI)=0.972, and root mean square error of approximation (RMSEA)=0.052—and model UGC—χ^2^/df=2.5, *P*<.001, CFI=0.966, TLI=0.959, and RMSEA=0.065. All fit indexes met the acceptable standards [90], indicating that the hypothesized models fit the data well and it was allowable to conduct the subsequent path analyses.

#### Hypothesis Testing

#### Assessment of Direct Effects

We conducted hierarchical multiple regression analyses to examine the hypothesized direct effects in our models. The standardized path coefficient and corresponding significance of each hypothesis are shown in Table 1. As the results indicate, the usefulness of IGC had positive effects on medication adherence attitude (β=.258; *P*<.001), perceived social support (β=.296; *P*<.001), and perceived value of antidepressants (β=.316; *P*<.001); the positivity of UGC also had positive effects on medication adherence attitude (β=.226; *P*=.001), perceived social support (β=.404; *P*<.001), and perceived value of antidepressants (β=.251; *P*<.001). Thus, hypotheses 1, 2, and 4 were supported. Moreover, in both model IGC and model UGC, perceived social support and perceived value of antidepressants were verified to have positive effects on medication adherence attitude (β>0; *P*<.001). Therefore, hypotheses 3 and 5 were supported as well.

**Table 1:** Results of direct effect examination.

Hypotheses			β		*P* value
Model IGC^a^ (n=353)					
	Hypothesis 1a: usefulness→medication adherence attitude	.258		<.001	
	Hypothesis 2a: usefulness→perceived social support	.296		<.001	
	Hypothesis 4a: usefulness→perceived value of antidepressants	.316		<.001	
	Hypothesis 3: perceived social support→medication adherence attitude	.245		<.001	
	Hypothesis 5: perceived value of antidepressants→medication adherence attitude	.696		<.001	
Model UGC^b^ (n=358)					
	Hypothesis 1b: positivity→medication adherence attitude	.226		.001	
	Hypothesis 2b: positivity→perceived social support	.404		<.001	
	Hypothesis 4b: positivity→perceived value of antidepressants	.251		<.001	
	Hypothesis 3: perceived social support→medication adherence attitude	.229		<.001	
	Hypothesis 5: perceived value of antidepressants→medication adherence attitude	.677		<.001	

^a^IGC: institution-generated content.

^b^UGC: user-generated content.

#### Assessment of Mediating Effects

Through the above analysis of direct effects, we confirmed that the independent variables in this study had significant relationships with the hypothesized mediating variables and the dependent variable. Therefore, the preconditions for testing the mediating effects were satisfied [91]. In accordance with the suggestion by Zhao et al [92], we used bootstrapping analysis (supported by SPSS version 26.0, PROCESS model 4; IBM Corp) to examine the mediating effects of perceived social support and perceived value of antidepressants. As the results in Table 2 show, all the 95% CIs did not include 0, suggesting that the indirect effects of the usefulness of IGC and positivity of UGC on attitude toward medication adherence through perceived social support or perceived value of antidepressants were statistically significant. Therefore, hypotheses 6 and 7 were supported.

**Table 2:** Results of mediating effect examination.

Hypotheses		β (SE; 95% CI)
Model IGC^a^ (n=353)		
	Hypothesis 6a: usefulness→perceived social support→medication adherence attitude	.066 (.0271; .0175-.1243)
	Hypothesis 7a: usefulness→perceived value of antidepressants→medication adherence attitude	.249 (.0564; .1403-.3638)
Model UGC^b^ (n=358)		
	Hypothesis 6b: positivity→perceived social support→medication adherence attitude	.092 (.0433; .0200-.1901)
	Hypothesis 7b: positivity→perceived value of antidepressants→medication adherence attitude	.204 (.0808; .0494-.3713)

^a^IGC: institution-generated content.

^b^UGC: user-generated content.

In addition, Table 2 reveals that the mediating role of the perceived value of antidepressants on the relationship between IGC usefulness or UGC positivity and attitude toward medication adherence (β>.20, 95% CI not including 0) seemed to be stronger than that of perceived social support (β<.10, 95% CI not including 0). To assess the significance of this difference in a more modest way, we used the following *Z* statistic:



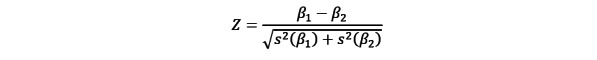



where β*_x_* are coefficients of the indirect effects and *s*^2^ are the squared SEs of the coefficients [93]. The results show that the indirect effect of IGC usefulness on attitude toward medication adherence via the mediation of perceived social support was significantly weaker than the indirect effect via the mediation of perceived value of antidepressants (*Z* statistic=–2.921∉[–1.96, 1.96]), whereas the difference was not salient for the indirect effects of UGC positivity (*Z* statistic=–1.227∈[–1.96, 1.96]).

#### Assessment of Moderating Effects

To exclude the possibility that hopelessness might be a direct influencing factor on the attitudes of patients with depression toward medication adherence, we conducted hierarchical multiple regression analyses to examine their relationship. Our conclusion was based on the findings of both model IGC (β=–.023; *P*=.68) and model UGC (β=–.023; *P*=.69). As a result, we can state with certainty that hopelessness is not a significant factor in shaping how patients with depression view medication adherence. To verify the moderating effects of hopelessness, we conducted a bootstrapping analysis (supported by SPSS version 26.0, PROCESS model 1) for the first step. As shown in Table 3, the interaction between usefulness of IGC and hopelessness was negatively related to attitude toward medication adherence (β=–.153, 95% CI –.2398 to –.0671; *P*<.001). Similar results were obtained for the interaction between positivity of UGC and hopelessness related to attitude toward medication adherence (β=–.135, 95% CI –.2246 to –.0446; *P*=.004). Therefore, hypothesis 8 was partially supported.

**Table 3:** Results of moderating effect examination.

Path and moderator variable				β (95% CI)		*P* value
Model IGC^a^ (n=353)						
	Usefulness → medication adherence attitude					
		Low hopelessness	.493 (.3211 to .6643)		<.001	
		Medium hopelessness	.322 (.1879 to .4564)		<.001	
		High hopelessness	.152 (–.0066 to .3098)		.06	
	Total effect—usefulness × hopelessness		−.153 (–.2398 to –.0671)		<.001	
Model UGC^b^ (n=358)						
	Positivity → medication adherence attitude					
		Low hopelessness	.450 (.2465 to .6533)		<.001	
		Medium hopelessness	.291 (.1249 to .4580)		<.001	
		High hopelessness	.133 (–.0582 to .3242)		.17	
	Total effect—positivity × hopelessness		−.135 (–.2246 to –.0446)		.004	

^a^IGC: institution-generated content.

^b^UGC: user-generated content.

**Figure 2:**
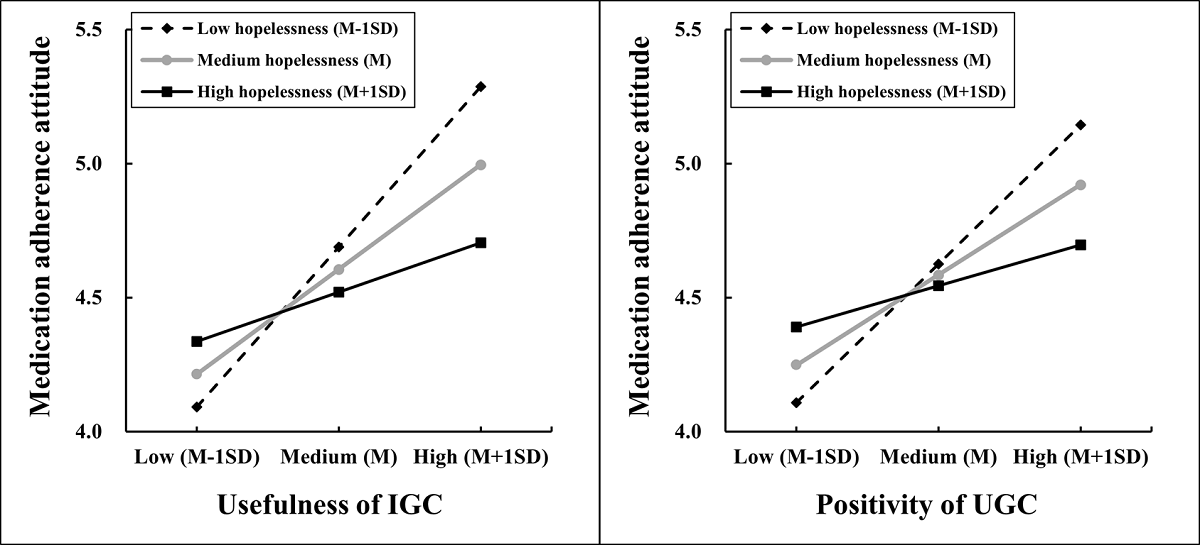
The effects of the interaction between the usefulness of institution-generated content (IGC) and hopelessness and the interaction between the positivity of user-generated content (UGC) and hopelessness on medication adherence attitude. M: mean; M-1SD: 1 SD below the mean; M+1SD: 1 SD above the mean.

To better present the nature of the moderating effects, we adopted the procedures by Aiken et al [94] for computing slopes 1 SD above and below the means of hopelessness and the slopes on the means of hopelessness to plot the interactions. As shown in Figure 2, with the hopelessness level increasing from low to high, the slopes became more and more steady, indicating that the effects of IGC usefulness and UGC positivity on attitude toward medication adherence were declining. Moreover, from Table 3, we can see that, with the conditions of low or medium hopelessness, the positive effects of IGC usefulness and UGC positivity on medication adherence attitude were all significant (*P*<.001, 95% CI not including 0), whereas with the condition of high hopelessness, the effects became insignificant (*P*=.06 for model IGC and *P*=.17 for model UGC, 95% CI including 0). This finding indicates that the significant positive influence of the usefulness of IGC and the positivity of UGC on attitude toward medication adherence can be eliminated by a high level of hopelessness.

In summary, hopelessness was verified as negatively moderating the effects of the usefulness of IGC and positivity of UGC on attitude toward medication adherence such that the positive effects of IGC usefulness and UGC positivity on attitude toward medication adherence strengthened when patients with depression had a low level of hopelessness but gradually diminished and even disappeared as the hopelessness level continued to increase. Therefore, hypothesis 8 was fully supported.

### Results of Robustness Check A

#### Assessment of Model Fit

We adopted a methodology similar to that of the main study to evaluate the study’s soundness in terms of model fit. We used the algorithm by Rogers and Schmitt [89] to parcel the scales of perceived social support and hopelessness into 3 items each. The modified model’s fit analysis yielded the following results: χ^2^/df=2.4, *P*<.001, CFI=0.937, TLI=0.925, and RMSEA=0.073. All fit indexes met the acceptable criteria [90], indicating that the hypothesized model fit well enough for follow-up path analyses.

#### Hypothesis Testing

We conducted hierarchical multiple regression analyses to examine the hypothesized direct effects. In addition, to rigorously compare the differences between the parallel hypotheses, we used the following *Z* statistic:



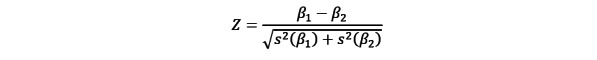



where β*_x_* are standardized coefficients and *s*^2^ are the squared SEs of the coefficients [93]. The results are shown in Table 4. All hypotheses were statistically significant (*P*<.01), ensuring the robustness of our findings and showing that useful IGC and positive UGC had simultaneous positive effects on attitudes toward medication adherence in patients with depression. Perceived social support was confirmed to have a weaker impact on attitude toward medication adherence than perceived value of antidepressants (*Z* statistic=–4.748∉[–1.96, 1.96]), whereas the differences between other parallel hypotheses were not statistically significant (*Z* statistic∈[–1.96, 1.96]).

**Table 4:** Results of direct effect examination of model IGC+UGC (n=270).

Hypotheses and comparison		β (SE)	*P* value	*Z* statistic
Hypothesis 1a: usefulness of IGC^a^→medication adherence attitude		.273 (.082)	<.001	—^b^
Hypothesis 1b: positivity of UGC^c^→medication adherence attitude		.247 (.087)	<.001	—
	Comparison: hypothesis 1a and hypothesis 1b	—	—	0.217
Hypothesis 2a: usefulness of IGC→perceived social support		.279 (.078)	<.001	—
Hypothesis 2b: positivity of UGC→perceived social support		.415 (.074)	<.001	—
	Comparison: hypothesis 2a and hypothesis 2b	—	—	–1.265
Hypothesis 4a: usefulness of IGC→perceived value of antidepressants		.290 (.064)	<.001	—
Hypothesis 4b: positivity of UGC→perceived value of antidepressants		.225 (.070)	.002	—
	Comparison: hypothesis 4a and hypothesis 4b	—	—	0.685
Hypothesis 3: perceived social support→medication adherence attitude		.254 (.067)	<.001	—
Hypothesis 5: perceived value of antidepressants→medication adherence attitude		.681 (.060)	<.001	—
	Comparison: hypothesis 3 and hypothesis 5	—	—	–4.748

^a^IGC: institution-generated content.

^b^Not applicable.

^c^UGC: user-generated content.

We used bootstrapping analyses (supported by SPSS version 26.0, PROCESS) to verify the hypothesized mediating and moderating effects. We present the bootstrapping results in Multimedia Appendices 6 and 7. All hypotheses were statistically significant, strengthening the robustness of our research outcomes.

### Results of Robustness Check B

#### Sample Characteristics

The approximate ODC use duration varied from 2 to 795 days among participants in model IGC-B and from 2 to 1268 days among participants in model UGC-B. ODC use frequency reported by participants in both models ranged from once a month (counted as 0.0333 visits per day) to at least once a day (counted as 1 visit per day). The rich diversity of the participants in terms of ODC use situations allowed the following effect examination to be informative. Moreover, as shown in Table 5, the participants recruited for this study covered all categories of ODC use situations. Therefore, we believe that the collected data for robustness check B fit the statistical requirements and study objective well.

**Table 5:** Participants’ online depression community use situations.

Duration^a^			Frequency^b^		Participants, n (%)
Model IGC^c^ -B (n=266)					
	Low	Low		21 (7.9)	
	Low	Medium		26 (9.8)	
	Low	High		10 (3.8)	
	Medium	Low		31 (11.7)	
	Medium	Medium		70 (26.3)	
	Medium	High		24 (9)	
	High	Low		29 (10.9)	
	High	Medium		43 (16.2)	
	High	High		12 (4.5)	
Model UGC^d^ -B (n=268)					
	Low	Low		27 (10.1)	
	Low	Medium		33 (12.3)	
	Low	High		15 (5.6)	
	Medium	Low		29 (10.8)	
	Medium	Medium		28 (10.4)	
	Medium	High		13 (4.9)	
	High	Low		50 (18.7)	
	High	Medium		52 (19.4)	
	High	High		21 (7.8)	

^a^Low=<1 month; medium=1 to 6 months; high=>6 months.

^b^Low=<1 visit per week; medium=1 to 4 visits per week; high=>4 visits per week.

^c^IGC: institution-generated content.

^d^UGC: user-generated content.

#### Hypothesis Testing

Hierarchical multiple regression analyses were conducted as the first step for examining the influence of ODC use situations. The standardized path coefficient and corresponding significance are shown in Table 6. As the results show, the direct effects of IGC usefulness and UGC positivity on medication adherence attitude were significantly positive (β>0; *P*<.001), consistent with our hypotheses. The results of model IGC-B and model UGC-B collectively indicate that ODC use duration had no significant relationships with IGC usefulness, UGC positivity, and medication adherence attitude (*P*>.10). In model IGC-B, ODC use frequency showed significantly positive relationships with IGC usefulness and medication adherence attitude (β>0; *P*<.01). In model UGC-B, ODC use frequency showed no significant relationships with UGC positivity and medication adherence attitude (*P*>.10).

**Table 6:** Results of direct effect examination.

Path		β	*P* value
Model IGC^a^ -B (n=266)			
	Usefulness→medication adherence attitude	.281	<.001
	Duration→usefulness	.038	.41
	Duration→medication adherence attitude	.053	.35
	Frequency→usefulness	.168	<.001
	Frequency→medication adherence attitude	.159	.006
Model UGC^b^ -B (n=268)			
	Positivity→medication adherence attitude	.266	<.001
	Duration→positivity	–.010	.84
	Duration→medication adherence attitude	.002	.98
	Frequency→positivity	.028	.55
	Frequency→medication adherence attitude	.081	.17

^a^IGC: institution-generated content.

^b^UGC: user-generated content.

To further ascertain the role of ODC use situations in the influence of IGC usefulness and UGC positivity on mediation adherence attitude, SPSS (version 26.0, PROCESS model 2) bootstrapping analysis was implemented. As the results in Table 7 show, the effects of IGC usefulness and UGC positivity on medication adherence attitude were insignificant when duration and frequency were both low (*P*>.10, 95% CI including 0), whereas they became stronger with a higher ODC use duration and frequency. When duration and frequency were both high, the effects of IGC usefulness and UGC positivity on medication adherence attitude were strongly significant (*P*<.001, 95% CI not including 0). The interaction between IGC usefulness and ODC use duration and the interaction between UGC positivity and ODC use duration showed no significant relationships with medication adherence attitude (*P*>.10, 95% CI including 0), suggesting that longer ODC use duration could not soundly guarantee a stronger effect of IGC usefulness or UGC positivity. The effects of the interaction between IGC usefulness and ODC use frequency and the interaction between UGC positivity and ODC use frequency on medication adherence attitude were significantly positive (β>0, 95% CI not including 0; *P*<.05). This indicates that higher ODC use frequency could prominently enhance the effects of IGC usefulness and UGC positivity on the attitudes of patients with depression toward medication adherence.

**Table 7:** Results of interactive effect examination.

Path and duration				Frequency		β (95% CI)		*P* value
Model IGC^a^ -B (n=266)^b^								
	Usefulness → medication adherence attitude							
		Low	Low		.039 (–.2202 to .2989)		.77	
		Low	Medium		.206 (–.0273 to .4395)		.08	
		Low	High		.532 (.2371 to .8276)		<.001	
		Medium	Low		.090 (–.1387 to .3177)		.44	
		Medium	Medium		.256 (.0556 to .4570)		.01	
		Medium	High		.583 (.3084 to .8566)		<.001	
		High	Low		.243 (–.0175 to .5038)		.07	
		High	Medium		.410 (.1660 to .6538)		.001	
		High	High		.736 (.4185 to 1.0538)		<.001	
Model UGC^c^ -B (n=268)^d^								
	Positivity → medication adherence attitude							
		Low	Low		.121 (–.1115 to .3540)		.31	
		Low	Medium		.170 (–.0426 to .3830)		.12	
		Low	High		.426 (.1773 to .6742)		<.001	
		Medium	Low		.159 (–.0530 to .3714)		.14	
		Medium	Medium		.208 (.0171 to .3993)		.03	
		Medium	High		.464 (.2292 to .6983)		<.001	
		High	Low		.316 (.0179 to .6137)		.04	
		High	Medium		.365 (.0787 to .6508)		.01	
		High	High		.620 (.2906 to .9501)		<.001	

^a^IGC: institution-generated content.

^b^Total effect—usefulness × duration: β=.0006 (95% CI –.0003 to .0015) and *P*=.17; total effect—usefulness × frequency: β=.7612 (95% CI .3044-1.2180) and *P*=.001.

^c^UGC: user-generated content.

^d^Total effect—positivity × duration: β=.0003 (95% CI –.0002 to .0007) and *P*=.23; total effect—positivity × frequency: β=.4472 (95% CI .0454-.8490) and *P*=.03.

In summary, this robustness check revealed how ODC use situations impacted the value of ODC as a tool to improve the attitudes of patients with depression toward medication adherence. By capturing the nuances between participants with different ODC use situations, the results of this robustness check collectively reduced the possibility that the positive influence of IGC usefulness and UGC positivity on the medication adherence attitudes of patients with depression was spuriously significant. Therefore, the reliability of our findings was further enhanced.

## Discussion

### Principal Findings and Comparison With Prior Work

In this study, we show that useful IGC and positive UGC in ODCs have positive effects on the attitudes of patients with depression toward medication adherence, with the mediating roles of perceived social support and perceived value of antidepressants and the moderating effect of hopelessness. This study contributes to the theoretical canon in multiple ways.

First, we uncovered new insights about the potential value of ODCs, which extend beyond their direct effects on mental health. ODCs are widely acknowledged as being able to provide a variety of social support to participants, including informational support, emotional support, instrumental support, social network support, and esteem support [8-10,16,18]. Given these advantages of ODCs, researchers generally believe that ODCs are a promising tool for coping with depression. With this grounded assumption, prior work in studies on ODCs has extensively explored ODCs’ direct effects on the mental states of individuals experiencing depression. Their findings are rather encouraging. They revealed that ODC engagement can contribute to the improvement of various psychological factors related to depression, such as the sense of loneliness, feeling of being understood, self-efficacy, and well-being [8-10,20,40]. More directly, several studies have found that patients’ depressive symptoms decrease over their participation in ODCs [10,95,96], and the significance of this effect has been confirmed by Goodwin et al [10]. These results offer important insights about ODCs’ role in depression treatment, suggesting that ODCs have direct effects on ameliorating patients’ mental health and can serve as an informal method to treat depression. However, the potential value of ODCs as an adjunct to formal depression treatments such as medication has not yet been fully appreciated. This niche topic is theoretically important because it indicates the likelihood that ODCs can benefit patients’ recovery from depression in diversified ways. Apart from being a direct treatment tool, ODCs may also indirectly impact depression recovery by facilitating patients’ appropriate adoption of mental health care services and products. Several scholars have corroborated that participating in OHCs can aid in improving patient compliance in the treatment process [6,11-13], whereas such a benefit in the ODC setting and the field of depression treatment remains unexplored. This study was designed to address this gap, verifying ODCs’ positive influence on the perceived value of antidepressants and attitudes toward medication taking among individuals experiencing depression. Our findings provide empirical evidence that the value of ODCs extends beyond influencing the mental status of individuals with depression and serves as an effective complement to formal treatment.

Second, this study introduces the community content perspective into the research on OHCs’ value in improving patients’ medication adherence. Previous literature on this topic has explored how patients may be affected by the overall idiosyncrasies of OHCs and how their participation patterns impact their ability to benefit from the communities [6,11-13]. However, there is a lack of knowledge about how community content impacts patients’ attitudes toward medication adherence. To address this gap, our study borrowed ideas from SOR theory and relevant literature and extracted 2 specific types of ODC content, namely, useful IGC and positive UGC. Our findings reveal that the usefulness of IGC and positivity of UGC do positively relate to patients’ willingness to adhere to medications. This offers a fresh perspective on how OHCs can be used as an intervention to address patients’ nonadherence problem, thus enriching the existing knowledge base.

Third, this study adds to the understanding of the inner mechanism of OHCs’ influence on patients’ medication adherence. Wu et al [12] found that health care information in OHCs enhances patients’ trust in health care services and, thus, boosts their adherence intentionality. Lu [6] proved that perceived social support plays a significant mediating role in the relationship between patient health information–seeking behaviors and patient compliance. However, little is known about the difference between the mediation effects of the 2 factors: the value judgment of the treatment and the social capital obtained by patients. Our research outcomes ascertain that perceived value of antidepressants plays a stronger mediating effect in the relationship between IGC usefulness and medication adherence attitudes than perceived social support, whereas such a distinction does not appear in the relationship between UGC positivity and attitudes toward medication adherence. This finding demonstrates that the professional health care information within OHCs majorly affects patient compliance by adjusting their perception about the medication’s value, whereas patient-to-patient communications impact patients’ willingness to follow the treatment through a more mixed mechanism. Thus, this study enriches the knowledge about the inner pathway of the influence of OHCs on patients’ medication adherence attitudes.

Fourth, we found that there is a specific limitation to the positive impact of ODCs on patients with depression. Grounded on the feelings-as-information theory, we theorized that the feeling of hopelessness can moderate the relationship between positive external stimuli and medication adherence in patients with depression. Our study discovered that useful IGC and positive UGC are powerful stimuli for enhancing the medication-taking attitudes of patients with depression who remain optimistic. However, when patients lose hope about their lives and the future, they tend to believe that any endeavor to improve their condition is futile, thus undermining the positive effects of ODCs. This finding provides a fundamental boundary condition for the positive effects of ODCs, aiding in clarifying the scenarios in which ODCs can and cannot be adopted as a tool for boosting the adherence of patients with depression to medicinal treatments.

Finally, this study is a call to action for future research in the field of OHCs to emphasize the importance of carefully evaluating the target population’s uniqueness. In the treatment process of physiological maladies (eg, HIV and diabetes), the sense of hopelessness is generally believed as a direct reason leading to patients’ nonadherence to the medications [71,72], whereas our research outcomes reveal that hopelessness does not directly result in poor medication-taking behaviors among patients with depression. Rather, it serves as a moderator, impacting their processing style for the ODC stimuli and, thus, eliciting different response patterns in terms of medication adherence attitudes. This peculiar phenomenon is conceivably caused by the fact that the current public distrust of the value of antidepressants is prevalent [27,29]. Our findings indicate the significance of recognizing that the same variable can have a vastly differentiated influence mechanism among individuals with different diseases. When theorizing on the impact of OHCs on patients, close scrutiny with regard to the uniqueness of the diseases they experience and the realistic social backgrounds related to the diseases is necessary.

### Practical Implications

Our findings have salient practical implications. There is currently a large population of people with depression in China [1], and many of them exhibit poor medication-taking behaviors [2]. The Chinese government has recognized the magnitude of this problem and formulated policies to address it, such as the “Action Plan for Depression Prevention and Treatment” [97], in which the importance of online channels such as ODCs is emphasized. The results of this study confirm that ODCs can be an effective complementary approach to improving the attitudes of patients with depression toward medication adherence and provide meaningful guidelines for practitioners.

One the one hand, governmental bodies, hospitals, or other health-related institutions should ensure that the content they provide is helpful to users. More specifically, they need to certify the quality of the content they create. Inaccurate information that confuses or misleads users can lead them to take inappropriate actions in the therapeutic process [39]. We urge practitioners to determine what information is genuinely needed by patients with depression and produce high-quality content in response while also purging inaccurate or misleading content from their sites. In contrast, ODC administrators should endeavor to create positive atmospheres within the communities. Our study confirms that ODC content can serve as stimuli for patients and content that presents positive coping strategies can inspire and empower patients to combat their depression. However, negative content such as messages about suicide or self-injurious behaviors [16] is harmful, and administrators should establish guidelines that encourage users to share optimistic content and avoid comments that are offensive or pessimistic. With these efforts, ODCs’ positive effects on patients can be strengthened.

Meanwhile, the recommendations we make can also be selectively applied in certain other contexts as depression is a representative mood disorder and often manifests as a comorbidity in patients with other severe diseases [98]. Specifically, the findings of our research can help practitioners better understand how to use OHCs as a tool to enhance treatment adherence for patients with other mood disorders (eg, posttraumatic stress disorder) that have similar symptoms to those of depression or for patients with other illnesses that co-occur with depression or have depression as a comorbidity.

### Limitations and Future Research

This study has a few limitations and prospects. Above all, cross-sectional questionnaire survey method was used in this study. This led to several deficiencies. First, we could only measure patients’ attitudes toward medication adherence at the time of the investigation. We were unable to capture the dynamic changes in participants’ attitudes from a within-subject perspective. As a result, even though we had examined the distinctions between participants with different ODC use situations, we could not fully exclude the potential that the effects of IGC usefulness and UGC positivity on participants’ medication adherence attitudes were spuriously significant. Meanwhile, although the attitudes of patients with depression toward medication adherence are known to be significantly associated with their adherence performance [65], we cannot ensure that ODCs’ positive effects on medication adherence attitudes are long-lasting and affect actual medication-taking behaviors. Second, as this study used self-reported data to examine the hypothesized model, the reliability of our results might be subject to the quality of the responses that participants provided. Although we made multiple efforts regarding the study design, data collection, and data analyses to avoid this risk, the robustness of our findings still cannot be fully guaranteed. Therefore, we suggest that future researchers conduct a longitudinal study tracing participants’ changes in attitudes and objective behaviors to address these remaining concerns.

Moreover, we included patients with both diagnosed depression and self-reported depression in our samples. While this practice helped make our samples more representative of the overall population of individuals with depression, it may have introduced a degree of measurement inaccuracy as people with self-reported depression had no actual experience taking medications and so their answers were based solely on their subjective feelings about antidepressants. Although we set this factor as one of the control variables to reduce the severity of this interference, whether the improvement in their perceived value of antidepressants and medication adherence attitude can be maintained after they start to take the medication remains unexamined. Hence, we encourage future research to explore the deviation between the perceptions of and attitudes toward antidepressants of patients with depression before and after taking them.

Furthermore, we split ODC content into IGC and UGC in this research, but our comparison between their influence on patients with depression was inadequate due to methodological limitations. First, recall bias exists in questionnaire-based surveys and possibly weakened the preciseness of respondents’ perceptions of the ODC content. Second, the attributes of IGC and UGC investigated in this research were different—usefulness and positivity, respectively. Consequently, our results cannot rigorously reflect the difference between the influence of IGC and UGC on patients. In our view, the attributes and providers of ODC content are closely linked to the potential for patients to derive substantial benefits from ODC involvement. To attain a deeper and more accurate understanding of the variability of ODC or OHC content, we suggest that future researchers conduct randomized controlled trials to compare the effects of IGC and UGC with the same features on patients.

Finally, the respondents of our survey were all Chinese, and thus, they share a common cultural background. Influenced by Confucian thought for thousands of years, Chinese culture emphasizes that individuals should possess self-control and resilience [17], leading to depression being frequently misunderstood as a kind of sentimentality or sign of weakness in China. As a result, many patients with depression face discrimination and marginalization in society. Under this background, people with depression in China are likely to rely more on ODCs, where the communications are anonymous and emotional support can be accessed [8,40]. Given this cultural influence, the generalizability and external validity of our findings may be limited. Consequently, we recommend that future research re-examine similar models in different cultural contexts.

### Conclusions

Grounded on the SOR and feelings-as-information theories, this study proposed a research model to examine the relationship between ODC content and the attitudes of patients with depression toward medication adherence. The results corroborate findings that the attitudes of patients with depression toward medication adherence can be improved as an outcome of the increase in perceived social support and perceived value of antidepressants, initially contributed by the stimulation of positive ODC content (useful IGC and positive UGC). Thereby, this study offers a novel solution to the problem of poor medication adherence among patients with depression in China. Our findings also provide suggestions for health care practitioners to generate online content that precisely matches the informational needs of patients with depression and recommend that ODC service providers make more efforts to regulate the community atmosphere. Moreover, this study verifies that hopelessness serves as an essential boundary condition for the effect of positive ODC content on the medication adherence attitudes of patients with depression. This finding warns health care practitioners that ODC interventions cannot be used as the only approach to addressing the problem of poor medication taking among patients with severe depressive symptoms.

## Appendix

Multimedia Appendix 1 Sample recruitment advertisement.

Multimedia Appendix 2 Sample characteristics.

Multimedia Appendix 3 Survey questions.

Multimedia Appendix 4 Mean, SD, and Cronbach α values for all variables.

Multimedia Appendix 5 Convergent validity and discriminant validity.

Multimedia Appendix 6 Results of the mediating effect examination of model IGC+UGC (n=270).

Multimedia Appendix 7 Results of the moderating effect examination of model IGC+UGC (n=270).

## Abbreviations

AVE: average variance extracted
CFI: comparative fit index
CR: composite reliability
IGC: institution-generated content
ODC: online depression community
OHC: online health community
RMSEA: root mean square error of approximation
SOR: stimulus-organism-response
TLI: Tucker-Lewis index
UGC: user-generated content

## Data Availability

The datasets generated during and analyzed during this study are available from the corresponding author on reasonable request.
