# Supplementary material for: Online Depression Communities as a Complementary Approach to Improving the Attitudes of Patients With Depression Toward Medication Adherence: Cross-Sectional Survey Study
Source: J Med Internet Res. 2024 Nov 19;26:e56166. doi: 10.2196/56166 (PMC11615551; doi:10.2196/56166)
Supplement: Multimedia Appendix 1 [file jmir_v26i1e56166_app1.docx]

Multimedia Appendix 1. Sample recruitment advertisement.

**Chinese Version**

*一项关于在线抑郁社群的研究*

大家好! 我们是来自（中国）西南财经大学和（英国）爱丁堡大学的研究团队。

基于目前国内抑郁症患者总人数高达9500万但治疗率较低的现状，本团队正在进行一项关于在线抑郁社群的影响作用的调查研究。我们希望向您（们）了解一些信息。

如果您（1）正在经历抑郁症，并且（2）是一位在线抑郁症社群的用户，则您契合本研究的调查范围。

***备注1:*** 如果您处于以下几个状态之一，则您契合第一个纳入标准：

- （确诊抑郁）您接受过医院的专业诊断，确诊为抑郁症，至今尚未完全康复。
- （自评抑郁）您测量过抑郁自评量表，结果显示您很可能患有抑郁症，并且您觉得自己仍旧处于抑郁状态。
- （自评抑郁）您长期体验到抑郁症相关的症状（例如，经常性地感到悲伤、想要流泪、空虚或绝望)，并且您强烈认为自己可能患有抑郁症。

***备注2：***在线抑郁症社群是指专门给抑郁症治疗的利益相关者(例如，患者、医生和心理咨询师)讨论抑郁症相关话题所用的在线社群。

如果您符合我们的纳入标准并且愿意帮助我们，我们非常欢迎您填写随这条消息所附的问卷。如果您感到不适或出于任何其他原因，可以随时退出填写。本次研究过程中调查的所有数据都将严格保密，除作为研究用途外绝无外传。若您的答复显示您符合本研究的调研范围，您将得到10元人民币作为感谢。

我们真诚地希望得到您的同意、参与和支持，一起为抑郁症患者群体做出贡献！若您身边有其他抑郁症患者也在使用在线抑郁社群，并且您愿意将我们的问卷分享给他们，我们将非常感激！真挚感谢您的配合与支持!

**English Translation**

*A Study About the Influence of Online Depression Communities*

Hi everyone! We are a research team from the Southwestern University of Finance and Economics in China and the University of Edinburgh in the United Kingdom.

It is estimated that there are currently more than 95 million people suffering from depression in China, while people’s adoption rate of depression treatment is rather low. Given this background, we are conducting a survey study that explores the influence of online depression communities. We would love to hear the voices from you!

You are eligible for this study if: (a) you are currently experiencing depression; and (b) you are a user of online depression communities.

***Note 1:*** You satisfy the first inclusion criterion if you meet one of these conditions:

- (Diagnosed Depression) You have been officially diagnosed as a patient with depression by the hospital, and you have not yet recovered.
- (Self-reported Depression) You have been suggested to have a high likelihood of being depressed by a self-rating depression scale, and you believe that you are still in a depressive status.
- (Self-reported Depression) You have experienced depression-related symptoms (e.g., frequent feelings of sadness, tearfulness, emptiness, or hopelessness) for a rather long time, and you strongly believe that you may have depression.

***Note 2:*** Online depression communities refer to digital communities exclusive for stakeholders in depression treatment (eg, patients, physicians, and psychological counselors) to discuss depression-related topics.

If you fit these requirements and are interested in helping, you are very welcome to participate in our study by responding to the digital questionnaire attached with this message! You can quit the survey whenever you feel uncomfortable or for any other reason. All information that you provide to us will be kept strictly confidential and will only be used for research purposes. Eligible participants will receive 10 RMB as a reward.

We sincerely hope to get your consent, participation, and support. Let’s contribute to the community of depression sufferers together! We are also deeply grateful if you would love to share our questionnaire with your depressed peers who are also using online depression communities. Many thanks for your cooperation and support!
