# Supplementary material for: Online Depression Communities as a Complementary Approach to Improving the Attitudes of Patients With Depression Toward Medication Adherence: Cross-Sectional Survey Study
Source: J Med Internet Res. 2024 Nov 19;26:e56166. doi: 10.2196/56166 (PMC11615551; doi:10.2196/56166)
Supplement: Multimedia Appendix 2 [file jmir_v26i1e56166_app2.docx]

Multimedia Appendix 2. Sample characteristics.

|  |  | Main Study | | Robustness  Check A | Robustness Check B | |
| --- | --- | --- | --- | --- | --- | --- |
|  |  | Model IGC  (n=353) | Model UGC (n=358) | Model IGC+UGC (n=270) | Model IGC-B (n=266) | Model UGC-B (n=268) |
| Variables | Items | Participants, n (%) | Participants, n (%) | Participants, n (%) | Participants, n (%) | Participants, n (%) |
| Mental health condition | Diagnosed depression | 240 (68) | 239 (66.8) | 183 (67.8) | 176 (66.2) | 179 (66.8) |
|  | Self-report depression | 113 (32) | 119 (33.2) | 87 (32.2) | 90 (33.8) | 89 (33.2) |
| Sex | Male | 143 (40.5) | 145 (40.5) | 119 (44.1) | 129 (48.5) | 128 (47.8) |
|  | Female | 210 (59.5) | 213 (59.5) | 151 (55.9) | 137 (51.5) | 140 (52.2) |
| Age | <12 years old | 6 (1.7) | 3 (0.8) | 2 (0.7) | 1 (0.4) | 0 (0) |
|  | 12-18 years old | 112 (31.7) | 117 (32.7) | 84 (31.1) | 38 (14.3) | 44 (16.4) |
|  | 19-24 years old | 119 (33.7) | 134 (37.4) | 96 (35.6) | 124 (46.6) | 139 (51.8) |
|  | 25-30 years old | 77 (21.8) | 72 (20.1) | 61 (22.6) | 77 (28.9) | 65 (24.3) |
|  | 31-40 years old | 31 (8.8) | 24 (6.7) | 20 (7.4) | 23 (8.6) | 19 (7.1) |
|  | 41-50 years old | 7 (2) | 6 (1.7) | 5 (1.9) | 3 (1.2) | 1 (0.4) |
|  | >51 years old | 1 (0.3) | 2 (0.6) | 2 (0.7) | 0 (0) | 0 (0) |
| Education | High school degree or less | 131 (37.1) | 139 (38.8) | 101 (37.4) | 55 (20.7) | 68 (25.4) |
|  | Junior college degree | 75 (21.3) | 73 (20.4) | 48 (17.8) | 61 (22.9) | 58 (21.6) |
|  | Bachelor’s degree | 135 (38.2) | 136 (38) | 112 (41.5) | 144 (54.1) | 134 (50) |
|  | Master’s degree or above | 12 (3.4) | 10 (2.8) | 9 (3.3) | 6 (2.3) | 8 (3) |
| Family of origin | Two-parent family | 227 (64.3) | 246 (68.7) | 181 (67) | 162 (60.9) | 160 (59.7) |
|  | Single-parent family | 72 (20.4) | 55 (15.3) | 47 (17.4) | 51 (19.2) | 56 (20.9) |
|  | Recomposed family | 35 (9.9) | 43 (12) | 29 (10.8) | 33 (12.4) | 34 (12.7) |
|  | Left behind family | 14 (4) | 12 (3.4) | 6 (2.2) | 14 (5.2) | 15 (5.6) |
|  | Disinheritance | 5 (1.4) | 2 (0.6) | 7 (2.6) | 6 (2.3) | 3 (1.1) |
| Marital status | Single | 251 (71.1) | 250 (69.8) | 192 (71.1) | 186 (69.9) | 180 (67.1) |
|  | In love | 56 (15.9) | 70 (19.6) | 43 (15.9) | 36 (13.5) | 56 (20.9) |
|  | Married | 30 (8.5) | 27 (7.5) | 25 (9.3) | 35 (13.2) | 24 (9) |
|  | Divorced | 16 (4.5) | 11 (3.1) | 10 (3.7) | 9 (3.4) | 8 (3) |
