# Supplementary material for: Online Depression Communities as a Complementary Approach to Improving the Attitudes of Patients With Depression Toward Medication Adherence: Cross-Sectional Survey Study
Source: J Med Internet Res. 2024 Nov 19;26:e56166. doi: 10.2196/56166 (PMC11615551; doi:10.2196/56166)
Supplement: Multimedia Appendix 3 [file jmir_v26i1e56166_app3.docx]

Multimedia Appendix 3. Survey questions.

**Main study**

*Demographic Information*

1. Mental health condition: ◻Diagnosed depression ◻Self-reported depression
2. Sex: ◻Male ◻Female
3. Age:

◻<12 years old ◻12-18 years old ◻19-24 years old ◻25-30 years old

◻31-40 years old ◻41-50 years old ◻>51 years old

1. Education:

◻High school degree or less ◻Junior college degree

◻Bachelor’s degree ◻Master’s degree or above

1. Family of origin:

◻ Two-parent family ◻ Single-parent family ◻ Recomposed family

◻ Left behind family ◻ Disinheritance

1. Marital status:

◻ Single ◻ In love ◻ Married ◻ Divorced

*Measurement Instruments*

(7-point Likert: strongly disagree, disagree, somewhat disagree, neither agree nor disagree, somewhat agree, agree, strongly agree)

1. Perceived credibility

(1) There is no fake information contained in the IGC/UGC.

(2) The IGC/UGC does not deliberately conceal some important facts.

(3) There is no malicious intent contained in the IGC/UGC.

(4) The information contained in the IGC/UGC is believable.

1. Perceived usefulness of IGC *[For Model IGC participants only]*

(1) I have gained a lot of knowledge about depression and antidepressants from the IGC.

(2) The information about depression and the coping strategies for depression contained in the IGC is helpful to me.

(3) The information about antidepressants’ mechanisms, values and potential side-effects carried in the IGC is helpful to me.

1. Perceived positivity of UGC *[For Model UGC participants only]*

(1) The UGC is friendly and harmonious.

(2) The UGC conveys an optimistic attitude.

(3) The UGC has offered me a lot of comfort.

(4) The members of the ODC always listen to others and give responses carefully.

1. Perceived social support

(1) There is someone willing to listen to me when I am in need.

(2) There is someone with whom I could share my joys and sorrows.

(3) There is someone able to offer me suggestions or help when I am in need.

(4) There is someone able to offer me emotional comfort or support when I am in need.

(5) There is someone who can be a real source of comfort to me when I am in need.

(6) There is someone who is ready to provide me with help.

(7) I can count on someone when things go wrong.

(8) There is someone with whom I could discuss about my sufferings and distress.

(9) There is someone willing to help me make decisions.

(10) There is someone who really cares about me.

(11) There is someone able to understand me.

1. Perceived value of antidepressants

(1) Antidepressants can help me recover from depression.

(2) The side-effects of antidepressants are tolerable.

(3) Antidepressants can help reduce my pain.

(4) Antidepressants can help reduce the negative impacts of depression on my daily life.

(5) Antidepressants have an important role in depression treatment.

1. Medication adherence attitude

(1) I am determined to finish the full prescribed duration of medication if the doctor believes I need medical treatment.

(2) I am determined to follow the instructions to take the antidepressants on time if the doctor believes I need medical treatment.

(3) I am determined to take the antidepressants in accordance with the suggested dosage if the doctor believes I need medical treatment.

1. Hopelessness

(1) I have no hope and enthusiasm toward the future.

(2) I always want to give up because I can’t make things better for myself.

(3) When things are going badly, I can’t believe they will be better later.

(4) My future seems dark to me, and I can’t imagine what my life would be like in 10 years.

(5) I have no enough time to accomplish the things I most want to do.

(6) I don’t expect to succeed in what concerns me most in the future.

(7) I don’t expect to get more of the good things in life than the average person.

(8) I have bad luck, and I don’t expect it to get any better.

(9) I don’t believe my past experiences have prepared me well for my future.

(10) All I can see ahead of me is unpleasantness rather than pleasantness.

(11) I don’t expect to get what I really want.

(12) I have no confidence on my future, and I don’t believe I will get better in the future.

(13) Things just won’t work out the way I want them to.

(14) I never get what I want so it’s foolish to want anything,

(15) It is very unlikely that I will get any real satisfaction in the future.

(16) I will experience more bad times than good times in the future.

(17) There’s no use really trying to get something I want because I probably won’t get it.

**Robustness Check A**

*Demographic Information*

1. Mental health condition: ◻Diagnosed depression ◻Self-reported depression
2. Sex: ◻Male ◻Female
3. Age:

◻<12 years old ◻12-18 years old ◻19-24 years old ◻25-30 years old

◻31-40 years old ◻41-50 years old ◻>51 years old

1. Education:

◻High school degree or less ◻Junior college degree

◻Bachelor’s degree ◻Master’s degree or above

1. Family of origin:

◻ Two-parent family ◻ Single-parent family ◻ Recomposed family

◻ Left behind family ◻ Disinheritance

1. Marital status:

◻ Single ◻ In love ◻ Married ◻ Divorced

*Measurement Instruments*

(7-point Likert: strongly disagree, disagree, somewhat disagree, neither agree nor disagree, somewhat agree, agree, strongly agree)

1. Perceived credibility

(1) There is no fake information contained in the IGC/UGC.

(2) The IGC/UGC does not deliberately conceal some important facts.

(3) There is no malicious intent contained in the IGC/UGC.

(4) The information contained in the IGC/UGC is believable.

1. Perceived usefulness of IGC

(1) I have gained a lot of knowledge about depression and antidepressants from the IGC.

(2) The information about depression and the coping strategies for depression contained in the IGC is helpful to me.

(3) The information about antidepressants’ mechanisms, values and potential side-effects carried in the IGC is helpful to me.

1. Perceived positivity of UGC

(1) The UGC is friendly and harmonious.

(2) The UGC conveys an optimistic attitude.

(3) The UGC has offered me a lot of comfort.

(4) The members of the ODC always listen to others and give responses carefully.

1. Perceived social support

(1) There is someone willing to listen to me when I am in need.

(2) There is someone with whom I could share my joys and sorrows.

(3) There is someone able to offer me suggestions or help when I am in need.

(4) There is someone able to offer me emotional comfort or support when I am in need.

(5) There is someone who can be a real source of comfort to me when I am in need.

(6) There is someone who is ready to provide me with help.

(7) I can count on someone when things go wrong.

(8) There is someone with whom I could discuss about my sufferings and distress.

(9) There is someone willing to help me make decisions.

(10) There is someone who really cares about me.

(11) There is someone able to understand me.

1. Perceived value of antidepressants

(1) Antidepressants can help me recover from depression.

(2) The side-effects of antidepressants are tolerable.

(3) Antidepressants can help reduce my pain.

(4) Antidepressants can help reduce the negative impacts of depression on my daily life.

(5) Antidepressants have an important role in depression treatment.

1. Medication adherence attitude

(1) I am determined to finish the full prescribed duration of medication if the doctor believes I need medical treatment.

(2) I am determined to follow the instructions to take the antidepressants on time if the doctor believes I need medical treatment.

(3) I am determined to take the antidepressants in accordance with the suggested dosage if the doctor believes I need medical treatment.

1. Hopelessness

(1) I have no hope and enthusiasm toward the future.

(2) I always want to give up because I can’t make things better for myself.

(3) When things are going badly, I can’t believe they will be better later.

(4) My future seems dark to me, and I can’t imagine what my life would be like in 10 years.

(5) I have no enough time to accomplish the things I most want to do.

(6) I don’t expect to succeed in what concerns me most in the future.

(7) I don’t expect to get more of the good things in life than the average person.

(8) I have bad luck, and I don’t expect it to get any better.

(9) I don’t believe my past experiences have prepared me well for my future.

(10) All I can see ahead of me is unpleasantness rather than pleasantness.

(11) I don’t expect to get what I really want.

(12) I have no confidence on my future, and I don’t believe I will get better in the future.

(13) Things just won’t work out the way I want them to.

(14) I never get what I want so it’s foolish to want anything,

(15) It is very unlikely that I will get any real satisfaction in the future.

(16) I will experience more bad times than good times in the future.

(17) There’s no use really trying to get something I want because I probably won’t get it.

**Robustness Check B**

*Demographic Information*

1. Mental health condition: ◻Diagnosed depression ◻Self-reported depression
2. Sex: ◻Male ◻Female
3. Age:

◻<12 years old ◻12-18 years old ◻19-24 years old ◻25-30 years old

◻31-40 years old ◻41-50 years old ◻>51 years old

1. Education:

◻High school degree or less ◻Junior college degree

◻Bachelor’s degree ◻Master’s degree or above

1. Family of origin:

◻ Two-parent family ◻ Single-parent family ◻ Recomposed family

◻ Left behind family ◻ Disinheritance

1. Marital status:

◻ Single ◻ In love ◻ Married ◻ Divorced

*ODC Usage Situations*

1. When was the first time you visited an online depression community?
2. How is your average frequency of visiting online depression communities?

◻ Once a month ◻Twice a month ◻Three times a month

◻ Once a week ◻ Twice a week ◻Three times a week

◻ Four times a week ◻ Five times a week ◻Six times a week

◻ At least once a day

*Measurement Instruments*

(7-point Likert: strongly disagree, disagree, somewhat disagree, neither agree nor disagree, somewhat agree, agree, strongly agree)

1. Perceived credibility

(1) There is no fake information contained in the IGC/UGC.

(2) The IGC/UGC does not deliberately conceal some important facts.

(3) There is no malicious intent contained in the IGC/UGC.

(4) The information contained in the IGC/UGC is believable.

1. Perceived usefulness of IGC *[For Model IGC-B participants only]*

(1) I have gained a lot of knowledge about depression and antidepressants from the IGC.

(2) The information about depression and the coping strategies for depression contained in the IGC is helpful to me.

(3) The information about antidepressants’ mechanisms, values and potential side-effects carried in the IGC is helpful to me.

1. Perceived positivity of UGC *[For Model UGC-B participants only]*

(1) The UGC is friendly and harmonious.

(2) The UGC conveys an optimistic attitude.

(3) The UGC has offered me a lot of comfort.

(4) The members of the ODC always listen to others and give responses carefully.

1. Medication adherence attitude

(1) I am determined to finish the full prescribed duration of medication if the doctor believes I need medical treatment.

(2) I am determined to follow the instructions to take the antidepressants on time if the doctor believes I need medical treatment.

(3) I am determined to take the antidepressants in accordance with the suggested dosage if the doctor believes I need medical treatment.
