# Supplementary material for: Online Depression Communities as a Complementary Approach to Improving the Attitudes of Patients With Depression Toward Medication Adherence: Cross-Sectional Survey Study
Source: J Med Internet Res. 2024 Nov 19;26:e56166. doi: 10.2196/56166 (PMC11615551; doi:10.2196/56166)
Supplement: Multimedia Appendix 4 [file jmir_v26i1e56166_app4.docx]

Multimedia Appendix 4. Mean, SD, and Cronbach α values for all variables.

|  | | Main Study | | | | | | Robustness Check A | | | Robustness Check B | | | | | |
| --- | --- | --- | --- | --- | --- | --- | --- | --- | --- | --- | --- | --- | --- | --- | --- | --- |
|  | | Model IGC (n=353) | | | Model UGC (n=358) | | | Model IGC+UGC (n=270) | | | Model IGC-B (n=266) | | | Model UGC-B (n=268) | | |
| Variables | | Mean | SD | α^a^ | Mean | SD | α^a^ | Mean | SD | α^a^ | Mean | SD | α^a^ | Mean | SD | α^a^ |
| 1. Usefulness of IGC^b^ | | 4.82 | 1.21 | .85 |  |  |  | 4.94 | 1.02 | .81 | 4.96 | 1.06 | .80 |  |  |  |
| 2. Positivity of UGC^c^ | |  |  |  | 4.50 | 1.15 | .84 | 4.65 | 1.07 | .85 |  |  |  | 4.54 | 1.13 | .84 |
| 3. Perceived social support | | 4.13 | 1.27 | .93 | 4.05 | 1.36 | .95 | 4.24 | 1.22 | .94 |  |  |  |  |  |  |
| 4. Perceived value of antidepressants | | 4.46 | 1.20 | .88 | 4.52 | 1.20 | .90 | 4.65 | 1.04 | .87 |  |  |  |  |  |  |
| 5. Medication adherence attitude | | 4.59 | 1.39 | .93 | 4.59 | 1.40 | .94 | 4.73 | 1.27 | .90 | 4.58 | 1.35 | .88 | 4.36 | 1.34 | .89 |
| 6. Hopelessness | | 5.20 | 1.11 | .95 | 5.25 | 1.18 | .96 | 5.19 | 1.05 | .95 |  |  |  |  |  |  |
| 7. ODC^d^ usage duration^e^ | |  |  |  |  |  |  |  |  |  | 161.29 | 163.09 |  | 281.17 | 315.42 |  |
| 8. ODC^d^ usage frequency^f^ | |  |  |  |  |  |  |  |  |  | 0.35 | 0.30 |  | 0.31 | 0.32 |  |
| *Control variables* | |  |  |  |  |  |  |  |  |  |  |  |  |  |  |  |
|  | 9-a. Credibility of IGC^b^ | 4.64 | 1.20 | .88 |  |  |  | 4.71 | 1.14 | .86 | 4.84 | 1.06 | .83 |  |  |  |
|  | 9-b. Credibility of UGC^c^ |  |  |  | 4.38 | 1.31 | .91 | 4.57 | 1.15 | .87 |  |  |  | 4.30 | 1.31 | .89 |
|  | *Demographic information* |  |  |  |  |  |  |  |  |  |  |  |  |  |  |  |
|  | 10. Mental health condition^g^ | 1.32 | 0.47 |  | 1.33 | 0.47 |  | 1.32 | 0.47 |  | 1.34 | 0.47 |  | 1.33 | 0.47 |  |
|  | 11. Gender^h^ | 1.59 | 0.49 |  | 1.59 | 0.49 |  | 1.56 | 0.50 |  | 1.52 | 0.50 |  | 1.52 | 0.50 |  |
|  | 12. Age^i^ | 3.11 | 1.08 |  | 3.06 | 1.03 |  | 3.13 | 1.07 |  | 3.35 | 0.88 |  | 3.23 | 0.82 |  |
|  | 13. Education^j^ | 2.08 | 0.94 |  | 2.05 | 0.94 |  | 2.11 | 0.96 |  | 2.38 | 0.84 |  | 2.31 | 0.89 |  |
|  | 14. Family of origin^k^ | 1.58 | 0.92 |  | 1.52 | 0.87 |  | 1.56 | 0.95 |  | 1.69 | 1.03 |  | 1.68 | 0.97 |  |
|  | 15. Marital status^l^ | 1.46 | 0.83 |  | 1.44 | 0.76 |  | 1.46 | 0.81 |  | 1.50 | 0.85 |  | 1.48 | 0.78 |  |

^a^α: Cronbach alpha.

^b^IGC: institution-generated content.

^c^UGC: user-generated content.

^d^ODC: online depression community.

^e^Duration: “1” = 1 day; “2” = 2 days; and so on.

^f^Frequency: “0.0333” = 0.0333 visit/day (i.e. once a month); “0.0667” = 0.0667 visit/day (i.e. twice a month); “0.1” = 0.1 visit/day (i.e. three times a month); “0.1429” = 0.1429 visit/day (i.e. once a week); “0.2857” = 0.2857 visit/day (i.e. twice a week); “0.4286” = 0.4286 visit/day (i.e. three times a week); “0.5714” = 0.5714 visit/day (i.e. four times a week); “0.7143” = 0.7143 visit/day (i.e. five times a week); “0.8571” = 0.8571 visit/day (i.e. six times a week); “1” = 1 visit/day (i.e. at least once a day).

^g^Mental health condition: “1” = Diagnosed depression; “2” = Self-report depression.

^h^Gender: “1” = Male; “2” = Female.

^i^Age: “1” = Younger than 12 years old; “2” = 12-18 years old; “3” = 19-24 years old; “4” = 25-30 years old; “5” = 31-40 years old; “6” = 41-50 years old; “7” = Elder than 51 years old.

^j^Education: “1” = High school degree or less; “2” = Junior college degree; “3” = Bachelor’s degree; “4” = Master’s degree or above.

^k^Family of origin: “1” = Two-parent family; “2” = Single-parent family; “3” = Recomposed family; “4” = Left-behind family; “5” = Disinheritance/Orphan.

^l^Marital status: “1” = Single; “2” = In love; “3” = Married; “4” = Divorced.
