# Supplementary material for: Online Depression Communities as a Complementary Approach to Improving the Attitudes of Patients With Depression Toward Medication Adherence: Cross-Sectional Survey Study
Source: J Med Internet Res. 2024 Nov 19;26:e56166. doi: 10.2196/56166 (PMC11615551; doi:10.2196/56166)
Supplement: Multimedia Appendix 5 [file jmir_v26i1e56166_app5.docx]

Multimedia Appendix 5. Convergent validity and discriminant validity.

| Main Study: Model IGC (n=353) | | | | | | | | | | | |
| --- | --- | --- | --- | --- | --- | --- | --- | --- | --- | --- | --- |
| Variables | | CR^a^ | AVE^b^ | 1 | 2 | 3 | 4 | 5 |  | |  |
| 1. Usefulness | | 0.847 | 0.649 | *0.806* |  |  |  |  |  | |  |
| 2. Perceived social support | | 0.929 | 0.547 | 0.424 | *0.740* |  |  |  |  | |  |
| 3. Perceived value of antidepressants | | 0.886 | 0.612 | 0.446 | 0.418 | *0.782* |  |  |  | |  |
| 4. Medication adherence attitude | | 0.932 | 0.821 | 0.343 | 0.349 | 0.711 | *0.906* |  |  | |  |
| 5. Hopelessness | | 0.952 | 0.541 | 0.064 | -0.226 | -0.100 | -0.078 | *0.736* |  | |  |
| Main Study: Model UGC (n=358) | | | | | | | | | | | |
| Variables | | CR^a^ | AVE^b^ | 1 | 2 | 3 | 4 | 5 |  | |  |
| 1. Positivity | | 0.838 | 0.565 | *0.752* |  |  |  |  |  | |  |
| 2. Perceived social support | | 0.950 | 0.636 | 0.555 | *0.797* |  |  |  |  | |  |
| 3. Perceived value of antidepressants | | 0.908 | 0.667 | 0.399 | 0.464 | *0.817* |  |  |  | |  |
| 4. Medication adherence attitude | | 0.946 | 0.853 | 0.348 | 0.328 | 0.696 | *0.924* |  |  | |  |
| 5. Hopelessness | | 0.959 | 0.580 | -0.039 | -0.234 | -0.125 | -0.032 | *0.762* |  | |  |
| Robustness Check A: Model IGC+UGC (n=270) | | | | | | | | | | | |
| Variables | | CR^a^ | AVE^b^ | 1 | 2 | 3 | 4 | 5 | 6 | |  |
| 1. Usefulness of IGC^c^ | | 0.811 | 0.589 | *0.767* |  |  |  |  |  | |  |
| 2. Positivity of UGC^d^ | | 0.845 | 0.576 | 0.441 | *0.759* |  |  |  |  | |  |
| 3. Perceived social support | | 0.933 | 0.559 | 0.329 | 0.544 | *0.748* |  |  |  | |  |
| 4. Perceived value of antidepressants | | 0.867 | 0.570 | 0.387 | 0.379 | 0.483 | *0.755* |  |  | |  |
| 5. Medication adherence attitude | | 0.904 | 0.759 | 0.346 | 0.369 | 0.361 | 0.705 | *0.871* |  | |  |
| 6. Hopelessness | | 0.949 | 0.523 | 0.038 | 0.025 | -0.222 | -0.145 | -0.065 | *0.723* | |  |
| Robustness Check B: Model IGC-B (n=266) | | | | | | | | | | | |
| Variables | | CR^a^ | AVE^b^ | 1 | 2 |  |  |  |  | |  |
| 1. Usefulness | | 0.802 | 0.575 | *0.758* |  |  |  |  |  | |  |
| 2. Medication adherence attitude | | 0.879 | 0.707 | 0.427 | *0.841* |  |  |  |  | |  |
| Robustness Check B: Model UGC-B (n=268) | | | | | | | | | | | |
| Variables | | CR^a^ | AVE^b^ | 1 | 2 |  |  |  |  | |  |
| 1. Positivity | | 0.836 | 0.560 | *0.748* |  |  |  |  |  | |  |
| 2. Medication adherence attitude | | 0.889 | 0.728 | 0.368 | *0.853* |  |  |  |  | |  |
| The numbers in *italics* are the square roots of AVE, with the correlations below them. | | | | | | | | |  |  |  |

^a^CR: composite reliability.

^b^AVE: average variance extracted.

^c^IGC: institution-generated content.

^d^UGC: user-generated content.
