# Supplementary material for: Online Depression Communities as a Complementary Approach to Improving the Attitudes of Patients With Depression Toward Medication Adherence: Cross-Sectional Survey Study
Source: J Med Internet Res. 2024 Nov 19;26:e56166. doi: 10.2196/56166 (PMC11615551; doi:10.2196/56166)
Supplement: Multimedia Appendix 6 [file jmir_v26i1e56166_app6.docx]

Multimedia Appendix 6. Results of the mediating effect examination of model IGC+UGC (n=270).

| Hypotheses | Path | β (95% CI) |
| --- | --- | --- |
| Hypothesis 6a. | Usefulness of IGC^a^→ | .089 (.0329 to .1540) |
|  | perceived social support→ |  |
|  | medication adherence attitude |  |
| Hypothesis 6b. | Positivity of UGC^b^→ | .102 (.0137 to .2062) |
|  | perceived social support→ |  |
|  | medication adherence attitude |  |
| Hypothesis 7a. | Usefulness of IGC^a^→ | .246 (.1200 to .3707) |
|  | perceived value of antidepressants→ |  |
|  | medication adherence attitude |  |
| Hypothesis 7b. | Positivity of UGC^b^→ | .178 (.0293 to .3461) |
|  | perceived value of antidepressants→ |  |
|  | medication adherence attitude |  |

^a^IGC: institution-generated content.

^b^UGC: user-generated content.
