# Supplementary material for: Online Depression Communities as a Complementary Approach to Improving the Attitudes of Patients With Depression Toward Medication Adherence: Cross-Sectional Survey Study
Source: J Med Internet Res. 2024 Nov 19;26:e56166. doi: 10.2196/56166 (PMC11615551; doi:10.2196/56166)
Supplement: Multimedia Appendix 7 [file jmir_v26i1e56166_app7.docx]

Multimedia Appendix 7. Results of the moderating effect examination of model IGC+UGC (n=270).

| Path (IGC^a^) | Moderator variable | β (95% CI) | *P* value |
| --- | --- | --- | --- |
| Usefulness→ | Low hopelessness | .515 (.3145 to .7160) | <.001 |
| medication | Medium hopelessness | .346 (.1857 to .5065) | <.001 |
| adherence attitude | High hopelessness | .177 (-.0203 to .3741) | .08 |
| Total effect—usefulness × hopelessness | | -.161 (-.2724 to -.0489) | .005 |
| Path (UGC^b^) | Moderator variable | β (95% CI) | *P* value |
| Positivity→ | Low hopelessness | .500 (.2854 to .7146) | <.001 |
| medication | Medium hopelessness | .311 (.1419 to .4802) | <.001 |
| adherence attitude | High hopelessness | .122 (-.0810 to .3253) | .24 |
| Total effect—positivity × hopelessness | | -.179 (-.2959 to -.0629) | .003 |

^a^IGC: institution-generated content.

^b^UGC: user-generated content.
